# Treatment clinical trial – three types – for children with fluency disorders and stuttering

**DOI:** 10.1590/2317-1782/20212020264

**Published:** 2021-10-22

**Authors:** Nathalia dos Santos Fernandes de Ávila, Fabiola Juste, Julia Biancalana Costa, Claudia Regina Furquim de Andrade

**Affiliations:** 1 Programa de Pós-graduação em Ciências da Reabilitação, Departamento de Fisioterapia, Fonoaudiologia e Terapia Ocupacional, Faculdade de Medicina, Universidade de São Paulo – USP - São Paulo (SP), Brasil.; 2 Departamento de Fisioterapia, Fonoaudiologia e Terapia Ocupacional, Faculdade de Medicina, Universidade de São Paulo – USP - São Paulo (SP), Brasil.

**Keywords:** Stuttering, Speech, Children, Treatment, Clinical Trial

## Abstract

**Purpose:**

To present a treatment clinical trial, involving three types of treatment for chronic developmental stuttering (CDS), to verify whether they present indicators and sufficient information to establish an effective and safe benefit-risk relationship.

**Methods:**

The study included 252 children between 2 and 12 years old, who underwent assessment and treatment for CDS. Among the selected children, 93 met the established inclusion criteria. After obtaining the scores for the risk of CDS (Protocol for the Risk of Developmental Stuttering), all children were assessed according to their fluency profile and the severity level of stuttering. The children underwent treatment for CDS Green, Yellow and Red Programs. The treatment chosen for each child was based on the analysis of the risk for CDS.

**Results:**

All therapeutic programs presented positive results in the post-treatment assessment considering the analyzed parameters, with the exception of word repetition, sound prolongation at the end of words, and intrusion of sounds/word segments.

**Conclusion:**

The tested therapeutic programs – green, yellow, and red – were efficient for most of the participants. The direct intervention used in the Red Program was highly efficient in promoting fluent speech. This result suggests that for most of the patients with a higher risk of developing the chronic form of stuttering, the use of specific fluency promotion techniques is indicated.

## INTRODUCTION

Evidence-based medicine relies on scientific data to validate the use of a certain treatment. Although little used among speech therapists, especially in Brazil, the methodology of treatment clinical trials has been strongly encouraged internationally for quality control and effectiveness verification. A clinical trial of speech treatment is a planned experiment designed to assess the effectiveness of a given therapeutic process applied to a specific communication disorder, in the case of this study, chronic developmental stuttering (CDS)^(1)^.

According to the Diagnostic and statistical manual of mental disorders DSM-5, stuttering is a fluency disorder of neurodevelopmental origin that appears in the preschool years^(2)^ at the critical stage of emergence of the neural networks responsible for the development and stable control of motor speech processing^(3)^. The emergence and establishment of stuttering is predominantly of genetic origin, generating a complex and non-linear multifactorial interaction, which involves motor, linguistic, emotional, and psychosocial factors^(2-4)^.

The observable symptoms of CDS include involuntary disruptions of speech flow. There is a consensus that not spontaneously recoverable disruptions of speech are essential components of stuttering^(4,5)^. Recent imaging, structural and functional studies analyzing structural activities and connectivity in specific areas of the brain^(3,6-10)^ indicated the following consistent findings in children with stuttering, aged between 3 and 12 years:

- Reduced white matter development in the left oral motor region and reduced gray matter development in the left inferior frontal region (Broca)^1^;
- Reduced functionality and connectivity in the network: basal nuclei, thalamus, and cortex. This network is responsible for individual motion control. Children with stuttering also showed (similarly to adults with stuttering) reduced connectivity between networks involving auditory and motor interactions (superior temporal gyrus, left posterior, insula, supplementary motor area, and superior frontal gyrus).

In epidemiological terms, CDS is a universal disorder and no data specifying influence of language, race, or socioeconomic and cultural conditions have been reported. There is an evident gender variability of 3/1, that is, only one girl is affected for every three boys. The most common reason is found between incidence and prevalence rates is 4/1, which means that for every four children with speech fluency disorder, one progresses to chronic stuttering and three have spontaneous recovery^(11,12)^.

Population data indicate that in 95% of the stuttering cases, both the development and detection of the symptoms of disruptions occur abruptly between 2 and a half and 5 years of age^(4)^. Recent studies suggest that approximately 5% of the children present alterations in speech fluency at some point of their speech and language development^(11,12)^. Neither a diagnosis nor any speech procedure are applied to this group of children, leading to a sub-diagnosis of mild or very mild stuttering cases, which are not reported as spontaneously recovered stuttering.

Regarding child stuttering treatments, no consensus has been reached among different propositions. International and national systematic reviews on the topic indicate that only a few treatments have been effectively described. Neither nationally nor internationally there is no tendency to adopt treatments established through structured protocols based on solid scientific evidence^(13,14)^.

Evidence-based medicine for treatment studies follows a gold standard protocol, the Consolidated Standards of Reporting Trials (CONSORT), whose methodology is aimed at the assessment of medical treatments – epidemiological and including drug use. Initially, surgical (partly) and cognitive behavioral treatments were not addressed in this protocol. It is partially possible to apply the CONSORT regulations in the scope of clinical speech^(15,16)^.

The clinical trial research for the CDS treatment in this study also consolidates a proposal to assess clinically relevant outcome in speech therapy (especially the concept of progress). Although the outcome measures presented here were applied to the stuttering scope, the method can be used to other communication disorders, increasing our capacity for evidence-based scientific production^(17,18)^.

Since 1999, all children with CDS symptoms attended in the Laboratory of Speech Fluency Analysis, Facial Functions and Dysphagia of the Department of Physiotherapy, Speech Therapy and Occupational Therapy of the University of São Paulo School of Medicine, have been evaluated according to the protocol of chronic stuttering risk (PRGD). This protocol is composed of fifteen questions with three alternatives each (degrees of risk) ranging from characterization of the child to family background and social relations^(19)^. The application of the protocol is followed by an assessment of speech fluency^(20)^ and severity of the disorder^(21)^. The dataset supports the direction of the child to different therapeutic programs.

Our goal was to design a treatment clinical trial encompassing three categories to verify if the treatments tested presented indicators that allow to gather information for their application to continue, establishing an efficient, safe benefit-risk ratio.

## METHODS

This study was approved by the Research Ethics Committee of the Hospital das Clínicas of the University of São Paulo School of Medicine (CEP 2.235.170) and is characterized as a treatment clinical trial to investigate speech intervention based on outcome effect, dichotomous, with the number of stuttering disruptions pre- and post-CDS treatment as control variable^(15,16)^. It is considered a low-risk research and the continuity of the speech therapy was ensured for children who did not present positive scores at post-treatment. All legal caregivers signed a free and informed consent form.

### Participants

This study encompassed the analysis of 252 children, aged between 2 and 12 years, evaluated and treated for CDS. 93 of these children met all eligibility criteria, namely:

- Full identification information (name, date of birth, age, and name of the caregivers);
- Complete pre- and post-treatment assessments (PRGD^(19)^, Profile of Speech Fluency^(20)^ and SSI-3^(21)^);
- Speech Fluency Profile outside normal limits for age;
- Signature of Free and Informed Consent Form (FICF) at the time of service allowing the use of information;
- Absence of neurological and/or degenerative diseases and present tonal audiometry within normal limits;

### Material

After obtaining the scores of chronic stuttering risk (PRGD)^(19)^, all children were evaluated according to their speech fluency profile and degree of stuttering severity. The pre- and post-treatment analyses of speech were obtained based on the protocols of Speech Fluency Profile^(20)^ and Stuttering Severity Instrument *– 3* (SSI-3)^(21)^. The Green, Yellow, and Red Programs of CDS treatments were applied^(22)^.

### Procedures

#### Pre- and post-treatment

For the pre- and post-treatment assessments, we analyzed samples of spontaneous speech obtained using stimulus figure, accounting for 200 fluent syllables per participant. All analyses of Speech Fluency Profile and SSI-3 were applied^(20,21)^.

### Treatment

We determined the most indicated treatment for each child according to the analysis of degree of CDS risk, as follows: 19 children for the Green Program, 25 children for the Yellow Program, and 49 children for the Red Program. The Green Program involves indirect intervention and was indicated for the children with low risk of CDS. The Yellow Program uses mixed intervention and was recommended for the children with moderate risk of CDS. The Red Program is based on direct intervention and was assigned for the children with high risk of developing CDS^(22)^.

The indirect intervention program was established indirectly through three family counselling sessions focused on aspects of normal development of communication and speech fluency. As well as family linguistic aspects can either benefit or impair overcoming the current symptomatology, matters of the school scope and rights and duties of the stutterer are also addressed^(22)^.

The mixed intervention program was applied through indirect and direct interventions, in which both the child and family interact to promote and assist the protective communication of speech fluency. This program involves twelve sessions divided into four phases (experiment, stabilize, desensitize, and transfer) aiming to sensitize both the child and family about the benefits of soft, slow speech as a fundamental element of speech fluency^(22)^.

The direct intervention program was applied by interacting directly with the child and establishing specific adjustments regarding the family speech pattern. This program is carried out in twelve sessions and aims to provide resources and techniques to promote speech fluency and reduce speech disruptions and stuttering behavior^(22)^.

### Data analysis

For a clinical study of treatment outcome, it is highly recommended the blinding of the members in the team responsible for assessing the variables. In the case of this research, masking was used for the evaluators of speech fluency and stuttering severity, but not for the therapists responsible for the treatments. The evaluators responsible for analyzing the pre- and post-treatment speech samples were not aware of to which therapeutic program each participant belonged. To broaden the reliability of the study, 15% of the speech samples were subjected to peer-to-peer analysis (two speech therapists with experience in this type of analysis) reaching a level of agreement of 85% (k=0.48), indicating great agreement in the analysis of results.

#### Characterizing the participants

Characterization resulted in a total of 27 female participants with average age of 6.5 years (standard deviation 2.73), and 66 male participants with average age of 7.0 (standard deviation of 2.00). Thus, the distribution between genders was compatible with the literature reports^(11,12)^, indicating that the studied sample has generalization ability for the results for reflecting the actual scenario of the disorder distribution regarding gender and age.

#### Outcome variable

The outcome is the same alteration of a certain variable assessed at the beginning and in the end of the study. The outcome variable analyzed was the measure of stuttered syllables percentage, with an error margin of 0.25^(3,23,24)^.

#### Clinical outcome assessment

The assessments of the outcome effectiveness (variability of clinical condition under controlled conditions) referred to progress calculation^(17,18)^ (intragroup analysis) and variability of speech fluency profile^(20)^ and SSI-3^(21)^ (inter-group analysis).

## RESULTS

### Intragroup analysis

#### Progress calculation

The progress calculation^(17,18)^ is established individually through the relation between the result obtained in the pre-treatment (numerator) and the post-treatment result (denominator) for the percentage of stuttering disruptions. Such relation points to the progress factor that represents personal gain with the treatment. A progress index is considered positive above 1.25, and a negative index is below 0.75, while values between 0.76 and 1.24 indicate no variation. A percentage value of stuttering disruptions of 0 is considered as 0.1 to allow the safe calculation of the progress index.

Table 1 features the progress calculation for the Green Program. 12 (63.2%) of the 19 participants presented positive progress for the treatment, 6 (31.6%) had negative progress, and 1 (5.2%) showed result without variation.

**Table 1:** Progress calculation for the Green Program participants

Participants	% Stuttering disruptions		Progress factor	Progress index
	Pre-treatment	Post-treatment		
Subject 1	3.5	0.5	7	Positive
Subject 2	6.5	0.5	13	Positive
Subject 3	18	2.5	4.5	Positive
Subject 4	1.5	2.5	0.6	Negative
Subject 5	3	2	1.5	Positive
Subject 6	2	4.5	0.4	Negative
Subject 7	5	4.5	1.1	No variation
Subject 8	2	0.5	4.0	Positive
Subject 9	1	0.1	10.0	Positive
Subject 10	1.5	3	0.5	Negative
Subject 11	13	2	6.5	Positive
Subject 12	1.5	1	1.5	Positive
Subject 13	4.5	10.5	0.4	Negative
Subject 14	1	1.5	0.6	Negative
Subject 15	8	1	8.0	Positive
Subject 16	4	1	4.0	Positive
Subject 17	1.5	2.5	0.6	Negative
Subject 18	2	1.5	1.3	Positive
Subject 19	6.5	2	3.2	Positive

Table 2 presents the progress calculation for the Yellow Program. 17 (68%) of the 25 participants showed positive progress for the treatment, 5 (20%) had negative progress, and 3 (12%) presented no variation.

**Table 2:** Progress calculation for the Yellow Program participants

Participants	% Stuttering disruptions		Progress factor	Progress index
	Pre-treatment	Post-treatment		
Subject 1	4	2	2	Positive
Subject 2	16	3	5.3	Positive
Subject 3	1.5	1	1.5	Positive
Subject 4	4	1	4	Positive
Subject 5	9.5	5	1.9	Positive
Subject 6	0.1	0.1	1	No variation
Subject 7	1	0.1	10	Positive
Subject 8	9	0.5	18	Positive
Subject 9	0.1	0.5	0.2	Negative
Subject 10	1.5	0.1	15	Positive
Subject 11	3	6.5	0.4	Negative
Subject 12	1.5	0.1	15	Positive
Subject 13	4	0.1	40	Positive
Subject 14	1	0.5	2	Positive
Subject 15	8.5	1.5	5.6	Positive
Subject 16	8.5	5.5	1.5	Positive
Subject 17	4	0.1	40	Positive
Subject 18	10.5	6.5	1.6	Positive
Subject 19	9.5	0.1	95	Positive
Subject 20	0.5	1	0.5	Negative
Subject 21	2.5	3	0.8	No variation
Subject 22	0.5	0.5	1	No variation
Subject 23	0.5	2	0.25	Negative
Subject 24	3.5	0.1	35	Positive
Subject 25	0.1	1	0.1	Negative

Table 3 displays the progress calculation for the Red Program. 36 (73.5%) of the 49 participants presented positive progress for the treatment, 8 (16.3%) had negative progress, and 5 (10.2%) showed result without variation.

**Table 3:** Progress calculation for the Red Program participants

Participants	% Stuttering disruptions		Progress factor	Progress index
	Pre-treatment	Post-treatment		
Subject 1	1	2.5	0.4	Negative
Subject 2	14	4	3.5	Positive
Subject 3	3.5	0.5	7	Positive
Subject 4	6.5	4	1.6	Positive
Subject 5	17	2.5	6.8	Positive
Subject 6	8.5	2	4.2	Positive
Subject 7	10	2.5	4	Positive
Subject 8	12	4.5	2.7	Positive
Subject 9	5.5	0.1	55	Positive
Subject 10	1	6.5	0.1	Negative
Subject 11	11	1.5	7.3	Positive
Subject 12	6	5.5	1.1	No variation
Subject 13	2.5	3.5	0.7	Negative
Subject 14	35.5	12.5	2.8	Positive
Subject 15	2.5	0.1	25	Positive
Subject 16	6	2.5	2.4	Positive
Subject 17	2.5	1	2.5	Positive
Subject 18	4.5	3	1.5	Positive
Subject 19	7.5	4.5	1.7	Positive
Subject 20	5.5	12.5	0.4	Negative
Subject 21	2.5	4	0.6	Negative
Subject 22	6	4	1.5	Positive
Subject 23	1	1.5	0.7	Negative
Subject 24	4.5	5	0.9	No variation
Subject 25	2.5	0.5	5	Positive
Subject 26	11	6.5	1.6	Positive
Subject 27	12.5	5.5	2.2	Positive
Subject 28	4	1	4	Positive
Subject 29	6.5	0.5	13	Positive
Subject 30	2	1	2	Positive
Subject 31	7.5	0.5	15	Positive
Subject 32	5.5	12.5	0.4	Negative
Subject 33	3	2	1.5	Positive
Subject 34	6	4.5	1.3	Positive
Subject 35	3	2.5	1.2	No variation
Subject 36	3.5	2	1.7	Positive
Subject 37	11	0.1	110	Positive
Subject 38	29.5	8	3.6	Positive
Subject 39	6	1.5	4.3	Positive
Subject 40	6	3	2	Positive
Subject 41	11	2.5	4.4	Positive
Subject 42	3	3	1	No variation
Subject 43	4	2	2	Positive
Subject 44	3	3	1	No variation
Subject 45	3	0.1	30	Positive
Subject 46	11.5	2	5.7	Positive
Subject 47	5.5	0.5	11	Positive
Subject 48	25.5	17.5	1.4	Positive
Subject 49	1	1.5	0.7	Negative

### Intergroup analysis

The intergroups analysis considered the pre- and post-treatment results of the 93 participants, regardless of the type of treatment received. The data of speech fluency profile and SSI-3 were subjected to statistical analysis on the SPSS software, version 25. We performed descriptive analyses (average, standard deviation, median, minimum, and maximum) for all quantitative variables, as well as parametric inferential analysis comparing the pre-treatment and post-treatment results through t test for paired samples. For the qualitative variables, we conducted descriptive (total count and percentage) and inferential analyses comparing the pre-treatment and the post-treatment results through McNemar test. All analyses adopted the significance level of 5%.

Table 4 introduces the results of the speech fluency profile. Statistically significant differences appeared in the entire speech fluency profile, except for the category of common disfluency.

**Table 4:** Comparing the stages of the results for speech fluency profile of the participants

**Speech fluency profile**	**Stage**	**Average**	**Standard deviation**	**Minimum**	**Median**	**Maximum**	***p-value***
Number of common disfluency	pre	13.6	7.9	1.0	12.0	36.0	0.051
	post	11.9	7.8	0.0	10.0	42.0	
Number of stuttered disfluency	pre	12.4	12.1	0.0	8.0	71.0	<0.001^*^
	post	6.2	6.4	0.0	4.0	35.0	
Percentage of speech discontinuity	pre	13.1	8.3	1.5	11.0	42.5	<0.001*
	post	9.2	5.9	0.0	7.5	30.5	
Percentage of stuttered syllables	pre	6.2	6.1	0.0	4.0	35.5	<0.001*
	post	3.1	3.2	0.0		17.5	
Speech speed – words per minute	pre	69.5	28.0	12.8	67.6	142.2	0.031*
	post	76.1	24.4	31.8	73.9	151.4	
Velocage de speech – syllables per minute	pre	118.5	50.9	20.3	115.0	255.3	0.013*
	post	131.7	42.6	48.6	129.0	285.7	

* Significant difference according to t test for paired samples

Table 5 shows the pre- and post-treatment results for both common and stuttered disfluencies. Common disfluencies showed statistically significant difference for the type of word repetition, while stuttered disfluencies presented statistically significant difference for all variables, except for intrusions of sounds or segments and prolongations in the end of words.

**Table 5:** Comparing the stages of the results between common and stuttered disfluency

**Type of common disfluency**	**Stage**	**Average**	**Standard deviation**	**Minimum**	**Median**	**Maximum**	***p-value***
Hesitation	pre	4,2	4,5	0	3	18	0.168
	post	3,5	3,3	0	3	18	
Interjection	pre	1,2	1,9	0	0	8	0.235
	post	1,6	3,2	0	0	20	
Review	pre	1,2	1,4	0	1	6	0.728
	post	1,3	1,4	0	1	6	
Unfinished word	pre	0,5	0,9	0	0	5	0.929
	post	0,5	0,8	0	0	3	
Word repetition	pre	5,0	5,2	0	3	28	0.001^*^
	post	3,1	3,2	0	3	14	
Segment repetition	pre	1,3	1,5	0	1	6	0.179
	post	1,0	1,4	0	1	8	
Sentence repetition	pre	0,1	0,4	0	0	2	0.535
	post	0,1	0,3	0	0	2	
TOTAL	pre	13,6	7,9	1	12	36	0.051
	post	11,9	7,8	0	10	42	
Syllable repetitions	pre	2.2	2.6	0	1	11	<0.001*
	post	1.1	1.6	0	1	7	
Sound repetitions	pre	1.8	2.7	0	1	14	<0.001*
	post	0.7	1.3	0	0	6	
Extensions	pre	1.1	2.8	0	0	17	0.063
	post	0.5	1.4	0	0	10	
Blocks	pre	3.7	6.1	0	1	23	<0.001*
	post	1.4	3.0	0	0	21	
Pauses	pre	0.7	2.0	0	0	11	0.030*
	post	0.3	1.0	0	0	7	
Intrusion of sounds or segments	pre	0.5	1.6	0	0	8	0.052
	post	0.2	0.9	0	0	7	
TOTAL	pre	12.4	12.1	0	8	71	<0.001*
	post	6.2	6.4	0	4	35	

* Significant difference according to the t test for paired samples

By comparing the pre- and post-treatment results for SSI-3 in each assessment category, the t test for paired samples revealed statistically significant differences among the stages for all variables analyzed (frequency of disruptions – pre-treatment average: 9.6; post-treatment average: 6.3; p<0.001; duration of disruptions –pre-treatment average: 6.6; post-treatment average: 4.6; p<0.001; physical concomitants –pre-treatment average: 2.3; post-treatment average: 1.0; p<0.001; total score –pre-treatment average: 18.5; post-treatment average: 11.8; p<0.001).

## DISCUSSION

This is a blind, non-randomized study. The blind analysis of the treatments allowed no prior knowledge on the treatment received by the participant in the speech samples analysis. The participants were not randomized since their distribution in the different treatments was based on the indices of risk of chronic developmental stuttering (CDS). The assumption of the study was to verify the effectiveness of the different programs regarding their particularities.

This study is characterized as a clinical trial for meeting the following specific model: the treatments presented indicators that allow to gather information to continue with its application, establishing an efficient, safe benefit-risk ratio.

Because of the low scoring of CDS risk factors, the therapeutic approach for the Green Program – indirect intervention – focus on the relation family – child – communication. In this program, the child is indicated as disfluent when showing high spontaneous remission rate without specific intervention. The parents are involved in models of communication that enable speech fluency – lower emotional and linguistic impact of speech disruptions on the child, family, and school environments. The program is based on respecting the family dynamics without introducing any techniques and proved to be safe and efficient for 63.2% of the children.

For the Yellow Program – mixed intervention – the therapeutic approach is structured on a satisfactory prognosis for spontaneous recovery, despite the risk that speech disruptions become permanent. This program aims to improve the quality of the child’s communication, that is, allowing the child not to develop hesitation behaviors (fear of speaking out, shame, social isolation). Parents are guided to use basic techniques to promote speech fluency (reducing one’s own speech rate; simplifying sentences; establishing comfortable communicative shifts etc.). This program proved to be safe and efficient for 68% of the children.

The therapeutic approach for the direct intervention in the Red Program is structured on the fluent speech modeling with techniques and resources to reduce the number of stuttered disfluencies. This group of children has low index of spontaneous recovery of speech fluency and CDS prognosis. The program is based on the active engagement of the family by learning the specific techniques along with the child and contributing for the child to use them as much as possible. It proved to be safe and efficient for 73.5% of the children.

All therapeutic programs presented consistent results of improve in the post-treatment for the segments analyzed, except for word repetition, prolongations in the end of words, and intrusion of sounds/segments.

The findings of functional neuroimage obtained from the children with CDS point to lower functionality and connectivity of the neural networks involved in the production of fluent speech^(3,6-10)^. These results have been evident since the emergence of the symptomatology of typical stuttering disruptions. Due to such information, CDS treatments, especially in children, must be based on strong scientific evidence and prove to be safe and efficient according to solid theoretical basis. Considering the complexity of human communication, especially in natural context (real-time transmission of the message, within the specific linguistic regulations of each language, fluently and effortlessly), it is not expected that a given treatment is equally efficient for all patients, which would require the performance of controlled clinical trials.

All three treatment programs tested assume that we cannot treat all children with speech disorders in the same way. It is scientifically acknowledged that three indicators particularly point to high risk of CDS: heredity, male gender, and fixed articulating positions (blocking and repetitions of sounds/syllables)^(1,3,12,13,23-25)^. The remaining indicators derive from the integration recruitment (more or less efficient) of the auditory, linguistic, and sensorimotor systems. Communication ability is mediated, in all instances, by mental health and the social and family environments^(3,4,6-9)^.

The clinical trial presented met the three-fold criteria: evidence scientific (all three treatment programs are solidly based on extensive literature), patient’s information (based on the profile of CDS severity risk), and clinical expertise (blind therapists and evaluators with expertise in the area)^(3,6-10,15,16)^

It is worth considering the following limitations of this study: effect of sample size (the groups were homogeneous in terms of number of participants), non-randomization, and single variable control (%stuttered syllables). Additionally, the study did not assess other types of changes that can constitute stuttering (stuttering seasonal variability; speech outside the clinical situation; interaction abilities of the child; emotional and environmental profiles of the child, among others). Finally, this study did not address the long-term emergence of the disorder in children, thus hampering the analysis of permanent clinical condition outcome.

The scientific and social relevance of the study is especially associated with the large number of treated patients and the strict methodology of variable control. This study also demonstrates satisfactory and safe results for the child with symptomatology of chronic developmental stuttering regarding the treatment provided. There is little scientific evidence on the quality of the speech treatments offered either in Brazil or other countries^(18,25-27)^. It is essential to conduct studies to corroborate the treatment results. The importance of the treatment clinical trial proposed in this study refers not only to a better understanding on the effects of the tested treatments, but also to propose objective measures that can be applied to all communication disorders, either as monitoring or outcome indicators, using a numerically controllable variable (that should be considered in the specific constant attributes of each disorder).

## CONCLUSION

The therapeutic programs tested – Green, Yellow, and Red – proved to be efficient for most of the participants. The direct intervention, applied to the Red Program, was highly efficient at promoting fluent speech, indicating that for all cases of high chronicity index, specific techniques are recommended.

## Footnotes

1 The gray matter of the cortex is composed predominantly of the body of the neurons, while the white matter represents the myelinated fibers of the axons. The fibers are responsible for the communication link between neurons, without them there is no neural function.
