# ERRATUM: Treatment clinical trial – three types – for children with fluency disorders and stuttering

**DOI:** 10.1590/2317-1782/20212022208en

**Published:** 2023-02-10

Due to technical problems during the editorial production of the article “Treatment clinical trial – three types – for children with fluency disorders and stuttering” (DOI https://doi.org/10.1590/2317-1782/20212020264), published in CoDAS 2022;34(2):e20200264, the English version of this article was published with an error.

On page 8 of English version of the article, there should be an acknowledgements section with the following statement:

**ACKNOWLEDGEMENTS**

To CAPES, for promoting this research in the form of a Master's Scholarship.

