# Supplementary material for: Treatment clinical trial – three types – for children with fluency disorders and stuttering
Source: Codas. 2021 Oct 22;34(2):e20200264. doi: 10.1590/2317-1782/20212020264 (PMC9851192; doi:10.1590/2317-1782/20212020264)
Supplement: Translation

# Translation: Ensaio clínico de tratamento – em três modalidades – para crianças com distúrbios da fluência e gagueira

**Authors:** Nathalia dos Santos Fernandes de Ávila, Fabiola Juste, Julia Biancalana Costa, Claudia Regina Furquim de Andrade

**Affiliations:** 1 Programa de Pós-graduação em Ciências da Reabilitação, Departamento de Fisioterapia, Fonoaudiologia e Terapia Ocupacional, Faculdade de Medicina, Universidade de São Paulo – USP - São Paulo (SP), Brasil.; 2 Departamento de Fisioterapia, Fonoaudiologia e Terapia Ocupacional, Faculdade de Medicina, Universidade de São Paulo – USP - São Paulo (SP), Brasil.

**Keywords:** Gagueira, Fala, Crianças, Tratamento, Ensaio Clínico

## Abstract

**Objetivo:**

Delinear um ensaio clínico de tratamento – em três modalidades – que verificasse se os tramentos testados para a gagueira crônica do desenvolvimento (GCD) apresentam indicadores que permitam reunir informações para a continuidade da sua aplicação, estabelecendo uma relação benefício-risco eficaz e segura.

**Método:**

Para a realização do estudo foram analisadas 252 crianças, com idades entre 2 e 12 anos, que realizaram avaliação e tratamento para a GCD. Dentre as crianças atendidas, 93 cumpriram todos os critérios de elegibilidade. Após a obtenção dos escores de risco para GCD (Protocolo de Risco para a Gagueira do Desenvolvimento) todas as crianças foram avaliadas segundo seu perfil da fluência e grau de gravidade da gagueira. Foram aplicados os tratamentos para GCD: Programa Verde; Programa Amarelo e Programa Vermelho. A determinação do tratamento mais indicado para cada criança foi baseada na análise do grau de risco para a GCD.

**Resultados:**

Todos os programas terapeuticos apresentaram resultados de melhora pós-tratamento consistentes nos segmentos analisados com exceção de: repetição de palavras; prolongamentos no final das palavras e intrusão de sons/segmentos.

**Conclusão:**

Os programas terapêuticos testados – verde, amarelo e vermelho – foram eficientes para a ampla maioria dos participantes. A intervenção direta, aplicada no Programa Vermelho, foi altamente eficiente para a promoção da fala fluente, indicando que para os casos com maior índice de cronicidade a aplicação de técnicas específicas é indicada.

## INTRODUÇÃO

A prática baseada em evidências se apoia em dados científicos para validar o uso de um determinado tratamento. A metodologia dos ensaios clínicos de tratamento, embora pouco utilizada entre os fonoaudiólogos, principalmente brasileiros, vêm sendo fortemente incentivada internacionalmente, como forma de controle da qualidade e da verificação de eficácia dos tratamentos. Um ensaio clínico de tratamento fonoaudiológico é um experimento planejado e projetado para avaliar a eficácia de um determinado processo terapêutico aplicado a um dos distúrbios da comunicação, no caso do estudo aqui apresentado, à gagueira crônica do desenvolvimento (GCD)^(1)^.

Segundo o DSM-5, a gagueira é um distúrbio da fluência, de origem neuro-desenvolvimental, que surge nos anos pré-escolares^(2)^, na fase crítica de emergência das redes neurais que respondem pelo desenvolvimento e controle estável do processamento motor da fala^(3)^. O surgimento e desenvolvimento da gagueira é, predominantemente, de origem genética, gerando uma complexa e não linear interação multifatorial, que envolve fatores motores, linguísticos, emocionais e psicossociais^(2-4)^.

A GCD é um distúrbio cujos sintomas observáveis são as rupturas involuntárias no fluxo da fala. Há consenso que as rupturas na fala, não passíveis de recuperação espontânea, são os componentes essenciais da gagueira^(4,5)^. Os recentes estudos de imagens, estruturais e funcionais, que analisaram as atividades e conectividades estruturais em áreas específicas do cérebro^(3,6-10)^, indicaram que em crianças com gagueira, entre 3 e 12 anos, há achados consistentes de:

- Redução do desenvolvimento da substância branca na região motora oral esquerda e redução do desenvolvimento da substância cinzenta na região frontal inferior esquerda (Broca)^1^;
- Redução da funcionalidade e da conectividade na rede: núcleos da base, tálamo e córtex. Essa rede responde pelo controle dos movimentos individualizados. Nas crianças com gagueira também foram encontrados (assim como os achados em adultos com gagueira) uma redução da conectividade entre as redes envolvendo as interações auditiva e motoras (giro temporal, superior, posterior esquerdo, ínsula, área motora suplementar e giro frontal superior).

Em termos epidemiológicos, a GCD é um distúrbio universal, não sendo identificados dados que diferenciem a influência da língua, raça ou condição sócio-econômico-cultural. Há uma evidente variabilidade de gênero, sendo a razão dessa variabilidade 3/1, ou seja, a cada três meninos afetados há uma menina afetada. A razão comumente encontrada entre as taxas de incidência e prevalência é de 4/1, ou seja, a cada 4 crianças que apresentam o distúrbio da fluência, uma terá evolução para a gagueira crônica e três apresentarão recuperação espontânea^(11,12)^.

Há dados populacionais indicando que em 95% dos casos das gagueiras do desenvolvimento a detecção dos sintomas de rupturas se dá de forma abrupta entre 2 anos e meio e 5 anos de idade^(4)^. Estudos recentes sugerem que aproximadamente 5% das crianças apresentam, em algum momento do seu desenvolvimento da fala e linguagem, alterações da fluência da fala^(11,12)^. Esse grupo de crianças não recebe diagnóstico e/ou qualquer procedimento fonoaudiológico, havendo, portanto um sub diagnóstico das gagueiras de grau muito leve ou leve, que não são notificadas como gagueiras recuperadas de forma espontânea.

Em relação aos tratamentos para a gagueira infantil, não há consenso nas proposições. As revisões sistemáticas realizadas sobre o assunto (internacional e nacional) indicaram que poucos tratamentos são, efetivamente, descritos. Ainda não há tendência, nacional e internacional para a adoção de tratamentos estabelecidos através de protocolos estruturados e baseados em evidências científicas sólidas^(13,14)^.

A prática baseada em evidências para estudos de tratamento segue um protocolo, considerado o padrão ouro, que é o *Consolidated Standards of Reporting Trials* (CONSORT). A metodologia do CONSORT é direcionada para a avaliação de tratamentos médicos – epidemiológicos e com medicamentos. Os tratamentos cirúrgicos (parte deles) e os tratamentos cognitivo comportamentais não foram, inicialmente, contemplados nesse protocolo. A aplicação das normas CONSORT, na clínica fonoaudiológica são parcialmente possíveis de serem realizadas^(15,16)^.

A pesquisa do ensaio clínico de tratamento da GCD aqui apresentada consolida também, uma proposta de avaliação do desfecho clinicamente relevante em fonoaudiologia (principalmente o conceito de evolução). Embora as medidas de desfecho aqui apresentadas tenham sido aplicadas à gagueira, o método poderá ser usado para outros distúrbios da comunicação, aumentando a nossa capacidade de produção científica baseada em evidências^(17,18)^.

Desde 1999, todas as crianças com sintomas de GCD, atendidas no Laboratório de Investigação Fonoaudiológica da Fluência, Funções da Face e Disfagia do Departamento de Fisioterapia, Fonoaudiologia e Terapia Ocupacional da Faculdade de Medicina da Universidade de são Paulo, são inicialmente avaliadas por um protocolo para avaliação do risco para a gagueira crônica (PRGD). Esse protocolo é composto por quinze questões, com três alternativas (graus de risco) que abrangem desde os dados de caracterização da criança até seus antecedentes familiares e reações sociais^(19)^. Após a aplicação do protocolo é realizada a avaliação da fluência da fala^(20)^ e da gravidade do distúrbio^(21)^. O conjunto dos dados fundamenta o direcionamento da criança para os diferentes programas terapêuticos.

O objetivo do estudo foi delinear um ensaio clínico de tratamento – em três modalidades – que verificasse se os tramentos testados apresentam indicadores que permitam reunir informações para a continuidade da sua aplicação, estabelecendo uma relação benefício-risco eficaz e segura.

## MÉTODO

O estudo foi aprovado pelo Comitê de Ética em Pesquisa do Hospital das Clínicas da Faculdade de Medicina da Universidade de São Paulo (CEP 2.235.170) e caracteriza-se como um ensaio clínico de tratamento para investigação da intervenção fonoaudiológica com base no efeito de desfecho, dicotômico, cuja variável de controle foi o número de rupturas gagas pré e pós tratamento para a GCD^(15,16)^. A pesquisa é considerada de baixo risco e foi assegurada a continuidade da assistência fonoaudiológica para as crianças que não apresentaram escores positivos no pós-tratamento. Todos os responsáveis legais dos participantes assinaram o termo de consentimento livre e esclarecido. As crianças acima de 5 anos também o fizeram.

### Participantes

Para a realização do estudo foram analisadas 252 crianças, com idades entre 2 e 12 anos, que realizaram avaliação e tratamento para a GCD. Dentre as crianças atendidas, 93 cumpriram todos os critérios de elegibilidade, sendo:

- Apresentar dados de identificação completos (nome, data de nascimento, idade e nome dos responsáveis);
- Apresentar avaliação pré e pós-tratamento completas (PRGD^(19)^, Perfil da Fluência de Fala^(20)^ e SSI-3^(21)^);
- Apresentar Perfil da Fluência de Fala fora dos limites de normalidade para a idade;
- Apresentar Termo de Consentimento Livre e Esclarecido (TCLE), da época de realização do atendimento, assinado e permitindo a utilização das informações;
- Não apresentar doenças neurológicas e/ou degenerativas e apresentar audiometria tonal dentro dos limites da normalidade.

### Materiais

Após a obtenção dos escores de risco para a gagueira crônica (PRGD)^(19)^ todas as crianças foram avaliadas segundo seu perfil da fluência e grau de gravidade da gagueira. As análises de fala - pré e pós-tratamento - foram obtidas com base nos protocolos: Perfil da Fluência de Fala^(20)^ e *Severity Stuttering Instrument – 3* (SSI-3)^(21)^. Foram aplicados os tratamentos para GCD: Programa Verde; Programa Amarelo e Programa Vermelho^(22)^.

### Procedimentos

#### Avaliação pré e pós-tratamento

Para as avaliações pré e pós-tratamento foram analisadas amostras de fala espontânea, obtidas a partir de figura estímulo, contabilizando 200 sílabas fluentes para cada um dos participantes. Foram aplicadas todas as análises do Perfil da Fluência da Fala e do SSI-3^(20,21)^.

### Tratamento

A determinação do tratamento mais indicado para cada criança foi baseada na análise do grau de risco para a GCD. Foram indicadas 19 crianças para o Programa Verde, 25 crianças para o Programa Amarelo e 49 crianças para o Programa Vermelho. O Programa Verde é de intervenção indireta e foi indicado para as crianças com baixo risco para GCD. O Programa Amarelo é de intervenção mista e foi indicado para as crianças com risco para GCD. O Programa Vermelho é de intervenção direta e foi indicado para as crianças com alto risco para GCD desenvolvimento^(22)^.

O Programa de intervenção indireta foi desenvolvido de maneira indireta, por meio de três sessões de orientação familiar. O conteúdo das orientações possui enfoque em aspectos do desenvolvimento normal da comunicação e da fluência. Assim como os aspectos linguísticos familiares que podem auxiliar ou prejudicar a superação da sintomatologia atual, também são abordadas questões do âmbito escolar e dos direitos e deveres da pessoa que gagueja^(22)^.

O Programa de intervenção mista foi aplicado por meio da intervenção indireta e direta, onde criança e família interagem para a promoção e cuidados com a comunicação protetora da fluência. O Programa apresenta doze sessões divididas em quatro fases (Experimentar; Estabilizar; Dessensibilizar e Transferir), onde o objetivo é sensibilizar a criança e a família sobre os benefícios da fala suave e lentificada como elemento fundamental para a fluência^(22)^.

O Programa de intervenção direta foi aplicado através de intervenção direta com a criança e com ajustes específicos com os padrões de fala da família. O Programa é realizado em doze sessões e possui como objetivo oferecer recursos e técnicas para a promoção da fluência e a redução das rupturas de fala e dos comportamentos de gagueira^(22)^.

### Análise dos dados

Para um estudo clínico de desfecho de tratamento é altamente recomendável que haja o cegamento dos membros da equipe responsáveis pela aferição das variáveis. No caso da pesquisa realizada, os terapeutas responsáveis pelos tratamentos não foram mascarados. Houve o mascaramento dos avaliadores da fluência e da gravidade da gagueira. Os avaliadores responsáveis pelas análises das amostras de fala, pré e pós-tratamentos não conheciam à qual programa terapêutico o participante pertencia. Para ampliar a confiabilidade do estudo, 15% das amostras de fala foram submetidas à análise de dois juízes (dois fonoaudiólogos com experiência nesse tipo de análise). Foi obtido o nível de concordância de 85% (k=0,48) indicando alta concordância na análise dos resultados.

#### Caracterização dos participantes

Em relação à caracterização dos participantes para o gênero feminino houve o total de 27 participantes com idade média de 6,5 anos (desvio padrão 2,73). Para o gênero masculino houve o total de 66 participantes com idade média de 7,0 (desvio padrão de 2,00). Pode ser observado que a distribuição entre os gêneros foi compatível com os dados da literatura^(11,12)^, indicando que a amostra do estudo tem capacidade de generalização dos resultados por refletir a realidade de distribuição do distúrbio em relação ao gênero e idade.

#### Variável de desfecho

O desfecho é a mudança de uma determinada variável aferida, da mesma forma, no início e no final do estudo. A variável de desfecho analisada foi a medida da porcentagem de sílabas gaguejadas, com margem de erro variável de 0,25^(3,23,24)^.

#### Avaliação de desfecho clínico

As aferições de eficácia (variabilidade do quadro clínico sob circunstâncias controladas) do desfecho foram: cálculo de evolução^(17,18)^ (análise intragrupos) e variabilidade do perfil da fluência^(20)^ e do SSI-3^(21)^ (análise inter-grupos).

## RESULTADOS

### Análise intragrupos

#### Cálculo de evolução

O cálculo de evolução^(17,18)^ é estabelecido individualmente pela relação entre o resultado obtido no pré-tratamento (numerador) e o resultado pós-tratamento (denominador) para porcentagem de rupturas gagas. Essa relação indica o fator de evolução que representa o ganho pessoal com o tratamento. O índice de evolução foi considerado positivo acima de 1,25, negativo abaixo de 0,75 e sem variação entre 0,76 e 1,24. O valor de porcentagem de rupturas gagas igual a 0 foi considerado como 0,1 para permitir o cálculo seguro do índice de evolução.

Na Tabela 1 é apresentado o cálculo de evolução para o Programa Verde. Dos 19 participantes incluídos neste programa, 12 (63,2%) apresentaram evolução positiva para o tratamento; 6 (31,6%) apresentaram evolução negativa e 1 (5,2%) apresentou resultado sem variação.

**Tabela 1:** Cálculo de evolução para os participantes do Programa Verde

Participantes	% de rupturas gagas		Fator de evolução	Índice de evolução
	Pré-tratamento	Pós-tratamento		
Sujeito 1	3,5	0,5	7	Positivo
Sujeito 2	6,5	0,5	13	Positivo
Sujeito 3	18	2,5	4,5	Positivo
Sujeito 4	1,5	2,5	0,6	Negativo
Sujeito 5	3	2	1,5	Positivo
Sujeito 6	2	4,5	0,4	Negativo
Sujeito 7	5	4,5	1,1	Sem variação
Sujeito 8	2	0,5	4,0	Positivo
Sujeito 9	1	0,1	10,0	Positivo
Sujeito 10	1,5	3	0,5	Negativo
Sujeito 11	13	2	6,5	Positivo
Sujeito 12	1,5	1	1,5	Positivo
Sujeito 13	4,5	10,5	0,4	Negativo
Sujeito 14	1	1,5	0,6	Negativo
Sujeito 15	8	1	8,0	Positivo
Sujeito 16	4	1	4,0	Positivo
Sujeito 17	1,5	2,5	0,6	Negativo
Sujeito 18	2	1,5	1,3	Positivo
Sujeito 19	6,5	2	3,2	Positivo

Na Tabela 2 é apresentado o cálculo de evolução para o Programa Amarelo. Dos 25 participantes incluídos neste programa, 17 (68%) apresentaram evolução positiva para o tratamento, 5 (20%) apresentaram evolução negativa e 3 (12%) não apresentaram variação.

**Tabela 2:** Cálculo de evolução para os participantes do Programa Amarelo

Participantes	% de rupturas gagas		Fator de evolução	Índice de evolução
	Pré-tratamento	Pós-tratamento		
Sujeito 1	4	2	2	Positivo
Sujeito 2	16	3	5,3	Positivo
Sujeito 3	1,5	1	1,5	Positivo
Sujeito 4	4	1	4	Positivo
Sujeito 5	9,5	5	1,9	Positivo
Sujeito 6	0,1	0,1	1	Sem variação
Sujeito 7	1	0,1	10	Positivo
Sujeito 8	9	0,5	18	Positivo
Sujeito 9	0,1	0,5	0,2	Negativo
Sujeito 10	1,5	0,1	15	Positivo
Sujeito 11	3	6,5	0,4	Negativo
Sujeito 12	1,5	0,1	15	Positivo
Sujeito 13	4	0,1	40	Positivo
Sujeito 14	1	0,5	2	Positivo
Sujeito 15	8,5	1,5	5,6	Positivo
Sujeito 16	8,5	5,5	1,5	Positivo
Sujeito 17	4	0,1	40	Positivo
Sujeito 18	10,5	6,5	1,6	Positivo
Sujeito 19	9,5	0,1	95	Positivo
Sujeito 20	0,5	1	0,5	Negativo
Sujeito 21	2,5	3	0,8	Sem variação
Sujeito 22	0,5	0,5	1	Sem variação
Sujeito 23	0,5	2	0,25	Negativo
Sujeito 24	3,5	0,1	35	Positivo
Sujeito 25	0,1	1	0,1	Negativo

Na Tabela 3 é apresentado o cálculo de evolução para o Programa Vermelho. Dos 49 participantes incluídos neste programa, 36 (73,5%) apresentaram evolução positiva para o tratamento, 8 (16,3%) apresentaram evolução negativa e 5 (10,2%) apresentaram resultado sem variação.

**Tabela 3:** Cálculo de evolução para os participantes do Programa Vermelho

Participantes	% de rupturas gagas		Fator de evolução	Índice de evolução
	Pré-tratamento	Pós-tratamento		
Sujeito 1	1	2,5	0,4	Negativo
Sujeito 2	14	4	3,5	Positivo
Sujeito 3	3,5	0,5	7	Positivo
Sujeito 4	6,5	4	1,6	Positivo
Sujeito 5	17	2,5	6,8	Positivo
Sujeito 6	8,5	2	4,2	Positivo
Sujeito 7	10	2,5	4	Positivo
Sujeito 8	12	4,5	2,7	Positivo
Sujeito 9	5,5	0,1	55	Positivo
Sujeito 10	1	6,5	0,1	Negativo
Sujeito 11	11	1,5	7,3	Positivo
Sujeito 12	6	5,5	1,1	Sem variação
Sujeito 13	2,5	3,5	0,7	Negativo
Sujeito 14	35,5	12,5	2,8	Positivo
Sujeito 15	2,5	0,1	25	Positivo
Sujeito 16	6	2,5	2,4	Positivo
Sujeito 17	2,5	1	2,5	Positivo
Sujeito 18	4,5	3	1,5	Positivo
Sujeito 19	7,5	4,5	1,7	Positivo
Sujeito 20	5,5	12,5	0,4	Negativo
Sujeito 21	2,5	4	0,6	Negativo
Sujeito 22	6	4	1,5	Positivo
Sujeito 23	1	1,5	0,7	Negativo
Sujeito 24	4,5	5	0,9	Sem variação
Sujeito 25	2,5	0,5	5	Positivo
Sujeito 26	11	6,5	1,6	Positivo
Sujeito 27	12,5	5,5	2,2	Positivo
Sujeito 28	4	1	4	Positivo
Sujeito 29	6,5	0,5	13	Positivo
Sujeito 30	2	1	2	Positivo
Sujeito 31	7,5	0,5	15	Positivo
Sujeito 32	5,5	12,5	0,4	Negativo
Sujeito 33	3	2	1,5	Positivo
Sujeito 34	6	4,5	1,3	Positivo
Sujeito 35	3	2,5	1,2	Sem variação
Sujeito 36	3,5	2	1,7	Positivo
Sujeito 37	11	0,1	110	Positivo
Sujeito 38	29,5	8	3,6	Positivo
Sujeito 39	6	1,5	4,3	Positivo
Sujeito 40	6	3	2	Positivo
Sujeito 41	11	2,5	4,4	Positivo
Sujeito 42	3	3	1	Sem variação
Sujeito 43	4	2	2	Positivo
Sujeito 44	3	3	1	Sem variação
Sujeito 45	3	0,1	30	Positivo
Sujeito 46	11,5	2	5,7	Positivo
Sujeito 47	5,5	0,5	11	Positivo
Sujeito 48	25,5	17,5	1,4	Positivo
Sujeito 49	1	1,5	0,7	Negativo

### Análise intergrupos

Para a análise intergrupos foram considerados os resultados pré e pós tratamento os 93 participantes, independentemente do tipo de tratamento recebido. Os dados do perfil da fluência e do SSI-3 foram submetidos à análise estatística no software SPSS versão 25. Para as variáveis quantitativas, foram realizadas análises descritivas (média, desvio-padrão, mediana, mínimo e máximo) e inferenciais paramétricas comparando os resultados obtidos no pré-tratamento aos resultados obtidos no pós-tratamento por meio do teste t para amostras pareadas. Para as variáveis qualitativas, foram realizadas análises descritivas (contagem total e porcentagem) e inferenciais comparando os resultados obtidos pré-tratamento aos resultados obtidos pós-tratamento por meio do teste de McNemar. O nível de significância adotado em todas as análises foi de 5%.

A Tabela 4 apresenta os resultados do perfil da fluência da fala. Foram encontradas diferenças estatisticamente significativas no perfil da fluência da fala, com exceção da categoria de disfluências comuns.

**Tabela 4:** Comparação entre etapas dos resultados do perfil da fluência de fala apresentados pelos participantes da amostra

**Perfil da fluência**	**Etapa**	**Média**	**Desvio-padrão**	**Mínimo**	**Mediana**	**Máximo**	***p-value***
Número de disfluências comuns	pré	13,6	7,9	1,0	12,0	36,0	0,051
	pós	11,9	7,8	0,0	10,0	42,0	
Número de disfluências gagas	pré	12,4	12,1	0,0	8,0	71,0	<0,001*
	pós	6,2	6,4	0,0	4,0	35,0	
Porcentagem de descontinuidade de fala	pré	13,1	8,3	1,5	11,0	42,5	<0,001*
	pós	9,2	5,9	0,0	7,5	30,5	
Porcentagem de sílabas gaguejadas	pré	6,2	6,1	0,0	4,0	35,5	<0,001*
	pós	3,1	3,2	0,0		17,5	
Velocidade de fala – palavras por minuto	pré	69,5	28,0	12,8	67,6	142,2	0,031*
	pós	76,1	24,4	31,8	73,9	151,4	
Velocidade de fala – sílabas por minuto	pré	118,5	50,9	20,3	115,0	255,3	0,013*
	pós	131,7	42,6	48,6	129,0	285,7	

* Diferença significativa de acordo com o teste t para amostras pareadas

Na Tabela 5 são apresentados os resultados pré e pós tratamento para as disfluências comuns e gagas. Em relação às disfluências comuns, houve diferença estatisticamente significativa para a tipologia de repetição de palavras. Quanto às disfluências gagas, houve diferença estatisticamente significativa para a todas as variáveis com exceção das intrusões de sons ou segmentos e prolongamentos no final das palavras.

**Tabela 5:** Comparação entre etapas dos resultados para as disfluências comuns e gagas

**Tipologia das disfluências comuns**	**Etapa**	**Média**		**Desvio-padrão**	**Mínimo**	**Mediana**	**Máximo**	***p-value***
Hesitação	pré	4,2		4,5	0	3	18	0,168
	pós	3,5		3,3	0	3	18	
Interjeição	pré	1,2		1,9	0	0	8	0,235
	pós	1,6		3,2	0	0	20	
Revisão	pré	1,2		1,4	0	1	6	0,728
	pós	1,3		1,4	0	1	6	
Palavra não terminada	pré	0,5		0,9	0	0	5	0,929
	pós	0,5		0,8	0	0	3	
Repetição de palavras	pré	5,0		5,2	0	3	28	0,001*
	pós	3,1		3,2	0	3	14	
Repetição de segmento	pré	1,3		1,5	0	1	6	0,179
	pós	1,0		1,4	0	1	8	
Repetição de frases	pré	0,1		0,4	0	0	2	0,535
	pós	0,1		0,3	0	0	2	
TOTAL	pré	13,6		7,9	1	12	36	0,051
	pós	11,9		7,8	0	10	42	
Repetição de sílabas	pré		2,2	2,6	0	1	11	<0,001*
	pós		1,1	1,6	0	1	7	
Repetição de sons	pré		1,8	2,7	0	1	14	<0,001*
	pós		0,7	1,3	0	0	6	
Prolongamentos	pré		1,1	2,8	0	0	17	0,063
	pós		0,5	1,4	0	0	10	
Bloqueios	pré		3,7	6,1	0	1	23	<0,001*
	pós		1,4	3,0	0	0	21	
Pausas	pré		0,7	2,0	0	0	11	0,030*
	pós		0,3	1,0	0	0	7	
Intrusão de sons ou segmentos	pré		0,5	1,6	0	0	8	0,052
	pós		0,2	0,9	0	0	7	
TOTAL	pré		12,4	12,1	0	8	71	<0,001*
	pós		6,2	6,4	0	4	35	

* Diferença significativa de acordo com o teste t para amostras pareadas

Quanto ao SSI-3, foram comparados os resultados pré e pós-tratamento de cada uma das categorias da avaliação. Foram encontradas diferenças estatisticamente significativas de acordo com o teste t para amostras pareadas entre as etapas para todas as variáveis analisadas (frequência de rupturas – média pré: 9,6; média pós: 6,3; p<0,001; duração das rupturas – média pré: 6,6; média pós: 4,6; p<0,001; concomitantes físicos – média pré: 2,3; média pós: 1,0; p<0,001; escore total – média pré: 18,5; média pós: 11,8; p<0,001).

## DISCUSSÃO

O estudo realizado se caracterizou como cego, não randomizado. A análise cega dos tratamentos permitiu que não houvesse o conhecimento prévio do tratamento recebido pelo participante na análise das amostras de fala. Não houve randomização dos participantes, pois a distribuição dos mesmos nos diferentes tratamentos foi baseada nos índices de risco para a gagueira crônica do desenvolvimento (GCD). O pressuposto do estudo foi verificar a eficácia dos diferentes programas em suas especificidades.

O estudo se caracterizou como um ensaio clínico, pois responde ao paradigma específico: os tratamentos indicados apresentaram indicadores que permitiram reunir informação para a continuidade da sua aplicação, estabelecendo uma relação benefício-risco eficaz e segura.

Para o Programa Verde que é de intervenção indireta, em razão da baixa pontuação de fatores de risco para a GCD, a abordagem terapeutica é focada na relação família – criança – comunicação. Neste programa os indicativos são que a criança é disfluente com alta taxa de remissão espontânea sem intervenção específica. Os pais são envolvidos em modelos de comunicação facilitadores da fluência da fala – redução do impacto emocional e linguístico das rupturas da fala da criança na família e na escola. Esse programa é baseado no respeito à dinâmica familiar, sem a introdução de técnicas. O programa se mostrou seguro e eficaz para 63,2% das crianças.

Para o Programa Amarelo que é de intervenção mista, a abordagem terapeutica é estruturada no bom prognóstico para a recuperação espontânea, embora haja risco que as rupturas de fala se tornem permanentes. Neste programa, o foco é a melhor qualidade de comunicação da criança, ou seja, que a criança não desenvolva comportamentos de evitação (medo de falar, vergonha, isolamento social). Os pais são orientados a utilizar técnicas básicas que promovem a fluência da fala (redução da própria velociade de fala; simplificação de frases; estabelecimento de turnos comunicativos confortáveis, etc.). O programa se mostrou seguro e eficaz para 68% das crianças.

Para o Programa Vermelho que é de intervenção direta, a abordagem terapeutica é estruturada no modelamento de fala fluente com a demonstração de técnicas e recursos para reduzir o número de disfluências gagas. Para esse grupo de crianças, há baixo índice de recuperação espontânea da fluência e prognóstico indicativo de GCD. Neste programa, o envolvimento da família é ativo. A família aprende as técnicas específicas junto com a criança e contribui para que a criança possa usar as técnicas o maior número possível de vezes. O programa se mostrou seguro e eficaz para 73,5% das crianças.

Todos os programas terapeuticos apresentaram resultados de melhora pós-tratamento consistentes nos segmentos analisados, com exceção de: repetição de palavras; prolongamentos no final das palavras e intrusão de sons/segmentos.

Os achados de neuroimagem funcional obtidos com crianças com GCD indicam que há uma redução da funcionalidade e da conectividade nas redes neurais envolvidas na produção da fala fluente^(3,6-10)^. Esses achados são evidentes desde o início de surgimento das sintomatologias das rupturas típicas de gagueira. Em decorrência dessas informações há necessidade de que os tratamentos para a GCD, principalmente em crianças, tenham forte evidência científica. Os tratamentos devem ser seguros e eficientes, com sólida base teórica. Pela complexidade da comunicação humana, principalmente em contexto natural (transmissão da mensagem, em tempo real, dentro das normas linguísticas específicas de cada língua, de forma fluente e sem esforço) não é esperado que um mesmo tratamento possa ser igualmente eficiente para todos os pacientes. Para isso, é necessário que sejam feitos ensaios clínicos controlados.

Os três programas de tratamento testados partem do princípio que não podemos tratar da mesma forma todas as crianças que apresentam rupturas de fala. É reconhecido cientificamente que, principalmente, 3 indicadores são de alto risco para a GCD: hereditariedade, gênero masculino, posições articulatórias fixas (bloqueio e repetições de sons/sílabas)^(1,3,12,13,23-25)^. Os demais indicadores são decorrentes do recrutamento da integração (mais ou menos eficiente) dos sistemas auditivo, linguístico e sensório-motor. A habilidade de comunicação é mediada, em todas as instâncias, pela saúde mental e do ambiente familiar e social^(3,4,6-9)^.

O ensaio clinico apresentado cumpriu o tripé: evidência científica (os três programas de tratamento estão solidamente embasados em ampla literatura), característica do paciente (baseado no perfil da gravidade do risco para gagueira crônica) e expertise clínico (terapeutas e avaliadores cegos possuem expertise na área)^(3,6-10,15,16)^

O estudo apresenta limitações que devem ser consideradas tais como: efeito de tamanho da amostra (os grupos não eram homogêneos em termos do número de participantes; não randomização; controle de variável única (%sílabas gaguejadas) não tendo sido avaliadas as mudanças de outra natureza que possam compor a gagueira (variabilidade sazonal da gagueira; fala fora da situação clínica; habilidades interativas da criança; perfil emocional e ambiental da criança; entre outros). O estudo não contemplou o seguimento a longo prazo das crianças impedindo a análise de desfecho permanente do quadro clínico.

A relevância científica e social do estudo está vinculada, principalmente, ao grande número de pacientes tratados e à metodologia rígida de controle das variáveis. O estudo também demonstra há resultados bons e seguros para a criança, com sintomatologia para a gagueira crônica do desenvolvimento, em relação ao tratamento que lhe é oferecido. Há pouca evidência científica sobre a qualidade dos tratamentos fonoaudiológicos oferecidos tanto no Brasil quanto no exterior^(18,25-27)^. Estudos de confirmação dos resultados de tratamento são essenciais. A importância do ensaio clínico de tratamento aqui proposto é, além de contribuir para a compreensão de efeito dos tratamentos testados, a proposição de medidas objetivas possíveis de serem aplicadas a todos os distúrbios de comunicação, quer como índicadores de acompanhamento quer como indicadores de de desfecho, a partir de uma variável controlável numericamente (que deve ser considerada dentro dos atributos específicos constantes para cada um desses distúrbios).

## CONCLUSÃO

Os programas terapêuticos testados – verde, amarelo e vermelho – foram eficientes para a ampla maioria dos participantes. A intervenção direta, aplicada no Programa Vermelho, foi altamente eficiente para a promoção da fala fluente, indicando que para os casos com maior índice de cronicidade a aplicação de técnicas específicas é indicada.

## Footnotes

1 A substância cinzenta do córtex é composta, predominantemente, pelo corpo dos neurônios, enquanto a substância branca representa as fibras mielinizadas dos axônios. As fibras respondem pelo link de comunicação entre os neurônios, sem elas não há função neural.
