# Supplementary material for: ERRATUM: Treatment clinical trial – three types – for children with fluency disorders and stuttering
Source: Codas. 2023 Feb 10;35(1):e20220208. doi: 10.1590/2317-1782/20212022208en (PMC10010421; doi:10.1590/2317-1782/20212022208en)
Supplement: Translation

# Translation: ERRATA: Ensaio clínico de tratamento – em três modalidades – para crianças com distúrbios da fluência e gagueira

**DOI:** 10.1590/2317-1782/20212022208pt

Devido a problemas técnicos durante a produção editorial do artigo “Ensaio clínico de tratamento – em três modalidades – para crianças com distúrbios da fluência e gagueira” (DOI https://doi.org/10.1590/2317-1782/20212020264), publicado no periódico CoDAS 2022;34(2):e20200264, a versão em inglês deste artigo foi publicada com um erro.

Na página 8 da versão em inglês do artigo, deve haver uma seção de agradecimentos com a seguinte declaração:

**ACKNOWLEDGEMENTS**

To CAPES, for promoting this research in the form of a Master's Scholarship.
